# Comparative analysis of established and new biosensors for cyclic nucleotides

**DOI:** 10.1186/2050-6511-16-S1-A68

**Published:** 2015-09-02

**Authors:** Markus Milde, Martin Thunemann, Hariharan Subramanian, Katrin Ganzenberg, José Aparicio, Gülce Gülcüler, Viacheslav O Nikolaev, Robert Feil

**Affiliations:** 1 Interfakultäres Institut für Biochemie, University of Tübingen, Tübingen, Germany; 2 Institute of Experimental Cardiovascular Research, University Medical Center Hamburg-Eppendorf, Hamburg, Germany

## Background

Cyclic guanosine monophosphate (cGMP) and cyclic adenosine monophosphate (cAMP) are versatile second messengers relevant to many physiological and pathophysiological conditions. Live-cell imaging of these cyclic nucleotides with biosensors allows to elucidate their levels and dynamics under close-to-native conditions. However, in order to monitor cGMP and cAMP signals in parallel in the same cell, the respective biosensors must be spectrally compatible. We have characterised some commonly used as well as novel “green” and “red” biosensors for cGMP and cAMP with regard to their sensitivity and specificity.

## Methods

We used primary vascular smooth muscle cells (VSMCs) from mouse aortae to compare different cyclic nucleotide sensors. In intact VSMCs, cGMP was elevated by nitric oxide or natriuretic peptides and the signal/noise-ratio of each sensor and its sensitivity for each stimulator were analysed. To test the sensors’ sensitivity and specificity for cGMP *versus* cAMP, we permeabilised the cells with β-escin and applied defined concentrations of cyclic nucleotides. Furthermore, we cloned novel variants of the original CFP/YFP-based cytosolic cGi500 biosensor [1] including a membrane-targeted version termed “mcGi500” and a “red” variant termed “red cGi500” that contains the fluorophores tSapphire and Dimer2.

## Results

The fluorescence resonance energy transfer (FRET)-based ratiometric biosensor cGi500 turned out to be very reliable and suitable not only for cGMP imaging in cultured VSMCs, but also in living tissues of cGi500 transgenic mice [2] as assessed by spinning disk confocal FRET imaging. Confirming previous results by Russwurm and colleagues [1], the cGi500 sensor showed a good signal/noise-ratio, an EC_50_ value of 500 nM for cGMP, and a high selectivity for cGMP over cAMP ( 100-fold). Its membrane-targeted version, mcGi500, as well as the non-ratiometric cGMP sensor, FlincG3 [3], showed similar properties as cGi500. The “red” cGMP sensor, red cGi500, displayed similar properties as the “green” cGi500 and – at least in VSMCs – showed a better signal/noise-ratio than the previously described “red” cGMP sensor, red cGES-DE5 [4]. Additionally, we have characterised the “green” FRET-based cAMP sensor Epac1-camps [5] in VSMCs. This sensor showed a good signal/noise-ratio and its selectivity for cAMP over cGMP was 10 fold.

## Conclusion

Based on our comparative analyses, we conclude that the FRET-based ratiometric “green” cGi500 as well as its membrane-targeted version, mcGi500, and its “red” variant, red cGi500, should be useful tools for a broad spectrum of applications requiring real-time monitoring of cGMP signals. For example, mcGi500 could be used to visualise membrane-associated cGMP compartments and red cGi500 could be combined with the “green” Epac1-camps to study the crosstalk between cGMP and cAMP signalling in living cells and tissues.
